# Integrating transcriptional and protein interaction networks to prioritize condition-specific master regulators

**DOI:** 10.1186/s12918-015-0228-1

**Published:** 2015-11-14

**Authors:** Megha Padi, John Quackenbush

**Affiliations:** Department of Biostatistics and Computational Biology, Dana-Farber Cancer Institute, Boston, MA USA; Department of Medicine, Harvard Medical School, Boston, MA USA; Department of Biostatistics, Harvard School of Public Health, Boston, MA USA

**Keywords:** Network modeling, Gene regulation, Regulatory networks, Protein interaction networks, Cancer

## Abstract

**Background:**

Genome-wide libraries of yeast deletion strains have been used to screen for genes that drive phenotypes such as stress response. A surprising observation emerging from these studies is that the genes with the largest changes in mRNA expression during a state transition are not those that drive that transition. Here, we show that integrating gene expression data with context-independent protein interaction networks can help prioritize master regulators that drive biological phenotypes.

**Results:**

Genes essential for survival had previously been shown to exhibit high centrality in protein interaction networks. However, the set of genes that drive growth in any specific condition is highly context-dependent. We inferred regulatory networks from gene expression data and transcription factor binding motifs in *Saccharomyces cerevisiae*, and found that high-degree nodes in regulatory networks are enriched for transcription factors that drive the corresponding phenotypes. We then found that using a metric combining protein interaction and transcriptional networks improved the enrichment for drivers in many of the contexts we examined. We applied this principle to a dataset of gene expression in normal human fibroblasts expressing a panel of viral oncogenes. We integrated regulatory interactions inferred from this data with a database of yeast two-hybrid protein interactions and ranked 571 human transcription factors by their combined network score. The ranked list was significantly enriched in known cancer genes that could not be found by standard differential expression or enrichment analyses.

**Conclusions:**

There has been increasing recognition that network-based approaches can provide insight into critical cellular elements that help define phenotypic state. Our analysis suggests that no one network, based on a single data type, captures the full spectrum of interactions. Greater insight can instead be gained by exploring multiple independent networks and by choosing an appropriate metric on each network. Moreover we can improve our ability to rank phenotypic drivers by combining the information from individual networks. We propose that such integrative network analysis could be used to combine clinical gene expression data with interaction databases to prioritize patient- and disease-specific therapeutic targets.

**Electronic supplementary material:**

The online version of this article (doi:10.1186/s12918-015-0228-1) contains supplementary material, which is available to authorized users.

## Background

The sequencing of the yeast genome in the 1990s provided a catalog of genes, most of which were of unknown function. To functionally characterize the many newly discovered protein-coding genes, a genome-wide library was constructed of single gene deleted yeast strains, representing 96 % of all open reading frames [1]. This library was first used to screen for growth under stressful conditions such as high osmotic pressure, non-optimal glucose sources, or high pH, leading to the identification of distinct sets of driver genes responsible for growth under each of these conditions. Expression profiling of the same conditions revealed that the overlap between driver genes and differentially expressed genes was small and statistically insignificant [1–6]. This showed that the genes transcriptionally activated by a perturbation are not the same genes that drive phenotypic changes in response to that perturbation.

Systematic genome-wide deletion screens are now becoming possible in mammalian cells through the use of technologies such as TALENs and CRISPR-Cas9 [7–10]. However, it is infeasible to carry out such screens for every condition, drug, disease and patient. Characterizing multiple genetic perturbations would become exponentially complicated. As a consequence, such screens often fail to provide insight into why some genes are important and others are not. What is needed is a principled way to build models of driver genes in the context of their biological interactions.

Gene expression analysis, using expression arrays or RNA-sequencing, is the most widely used method to assay differences in cellular state between phenotypes. Robust statistics have been introduced to detect differential expression [11]. Clustering genes and looking for common binding sites upstream of their promoters can help identify transcription factors responsible for gene expression changes [12]. The Dynamic Regulatory Events Miner (DREM) is an example of a tool that searches for transcriptional regulators. DREM uses time-series expression data and physical interactions to find bifurcations in expression levels and the transcription factors responsible for them [13].

Network inference methods attempt to find coordinated patterns of expression, including the interactions between transcription factors (TFs) and genes. These methods are based on a wide variety of metrics, like pairwise correlation, linear regression, mutual information, classifiers and ordinary differential equations. Since TFs often act nonlinearly, through cooperativity or oligomerization, mutual information can perform better than linear models at detecting network interactions. Conditional mutual information has been used to infer more complex regulatory schemes [14, 15]. However, the most successful network inference methods combine the results of many algorithms or integrate information from sources other than gene expression [16]. A method called PANDA (Passing Attributes between Networks for Data Assimilation) explicitly models the activity of regulators and promoters by combining sequence motif data with gene expression through use of a message-passing algorithm [17]. The result of applying network inference methods is typically represented as a graph where the nodes represent the genes and the edges between them represent the presence of an interaction with likelihood above a specified threshold. The network edges can either be undirected, as in the case of a correlation based network, or directed, as in gene regulatory networks such as those produced by PANDA, where edges point from the regulator to their targets.

Protein-protein interaction (PPI) networks are used to represent physical binding events measured between protein pairs. In graphical representation, each node represents a protein and edges connect proteins that physically bind each other. The topology of PPI networks has been shown to reflect some properties of biological systems. The highly connected nodes, or high-degree “hubs,” in the yeast PPI network are enriched in essential genes that are lethal if deleted [18]. PPI networks have also been shown to possess a “community structure” that groups together proteins that interact more often with each other, and these communities are associated with common functions or biological processes [19]. It has been shown that disease genes are located close to each other in the human PPI network, and disease module proximity can be correlated with disease comorbidity [20, 21].

The problem with physical interaction networks is that they are difficult to measure in phenotype-specific contexts, so the PPI networks reflect an aggregate of likely networks, not accounting for whether proteins are expressed together in individual samples or disease states. In contrast, sequencing and gene expression profiling are flexible technologies allowing individual conditions or cellular states to be independently sampled. The networks inferred from sequence or expression data can thus carry information specific to the conditions of the experiment. However, fewer studies have examined how the topology of context-dependent networks can provide insight into critical genes driving individual phenotypes [22].

**Fig. 1:**
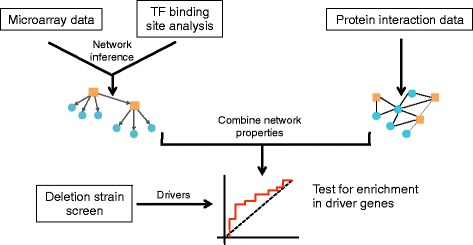
Workflow. Flowchart depicting how networks are constructed from different data types and combined to search for enrichment in driver genes

Here, we describe the construction and structural analysis of context-dependent and –independent networks in *Saccharomyces cerevisiae* and *Homo sapiens*, and the role of phenotypic drivers in these networks (Fig. 1). In yeast, we first analyzed data from an experiment designed to measure the transcriptional response of yeast to rapamycin, an antifungal drug that targets the Target of Rapamycin (TOR) pathway. We then investigated six other common stress perturbations in yeast: menadione, dithiothreitol (DTT), hydrogen peroxide, heat shock, diamide, and sorbitol. We found that the driver genes specific to each condition are often enriched among the central nodes of the transcriptional and protein interaction networks, and that combining the two networks increases the power to find drivers.

In humans, we performed a similar analysis on gene expression data from primary human fibroblasts perturbed by viral oncogenes, and looked for drivers of the viral-transformed phenotypes. We found that an integrative network analysis was able to enrich for cancer drivers that were not differentially expressed in the dataset. This suggests that combining data types to infer networks, and examining the structure of the resulting network, is a fruitful pipeline for analysis of transcriptional data.

## Results and discussion

### Differential expression is not a good predictor of drivers of rapamycin response

We first examined a dataset consisting of gene expression measured using the Affymetrix GeneChip Yeast Genome 2.0 Array platform in 97 strains of yeast upon exposure to rapamycin [23] (ArrayExpress accession: E-MTAB-412). The 97 strains consist of two parental strains called BY4716 and RM11-1a, and 95 haploid segregants that were created by crossing the parental strains. All the strains were profiled at six time points: 0, 10, 20, 30, 40, and 50 minutes. The data for all 97 strains and six time points were collectively normalized by the authors of [23] using robust multi-array average (RMA) normalization. Because we were interested in modeling expression changes due to rapamycin, we chose the strains that were the most sensitive to rapamycin. To do this we downloaded a separate dataset with measurements of the growth rates of the same yeast strains under various drug perturbations including rapamycin [24]. For each of the 79 strains that overlapped between the gene expression and growth rate datasets, we computed a score by taking the average of the growth rates (relative to growth under normal conditions) under the seven rapamycin-related conditions reported in [24]. We selected the 30 strains with a score of less than −0.1, corresponding to low growth rate in the presence of rapamycin. (The results of our analysis are robust to the choice of the growth rate cutoff; see Additional file 1: Supplementary Text and Figure S1 for more details.) We also collected a benchmark set of 396 experimentally validated driver genes that cause significantly altered growth under rapamycin from a separately published screen of a genome-wide deletion library [25].

**Fig. 2:**
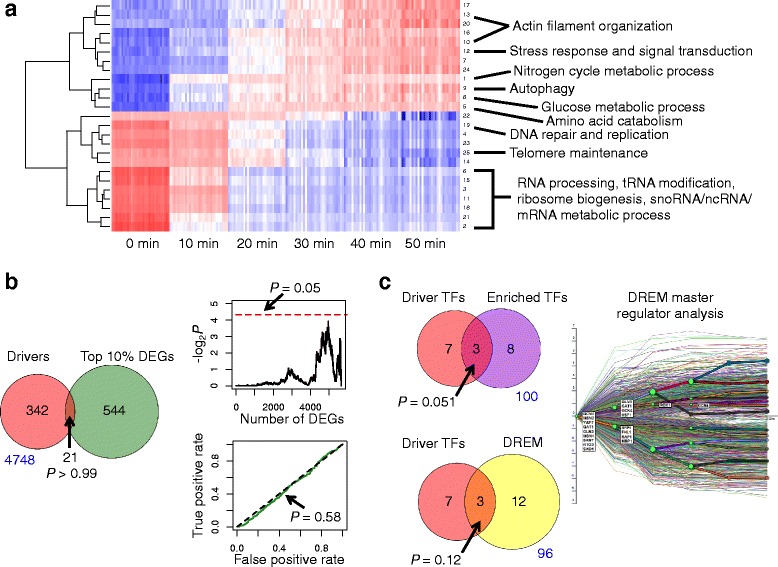
Standard gene expression analyses are not sufficient to identify drivers. **a** Heatmap depicting 25 gene expression clusters in 30 strains of rapamycin-sensitive yeast over six time points. Red and blue colors represent, respectively, positive and negative standardized expression (mean-subtracted and normalized by standard deviation in a row-wise manner) that has been averaged over the genes in each cluster. Examples of enriched GO terms are provided for selected clusters. Rows are labeled by cluster numbers as defined in Additional file 2. **b** Venn diagram shows the overlap between the top 10 % differentially expressed genes (DEGs) and the driver genes present on the array. *P*-value is from Fisher’s exact test. Number in blue denotes number of genes in universe not counted inside the Venn diagram. Top plot depicts the significance of the overlap as the number of DEGs is varied. Red dashed line indicates the minimum value for statistical significance. Bottom plot shows the receiver-operator characteristic (ROC) for the ranked list of DEGs and its overlap with driver genes. Dashed black line indicates expected ROC from random chance. *P*-value corresponds to a Kolmogorov-Smirnov test. **c** Venn diagrams indicating overlaps between driver transcription factors (TFs) and either the enriched TFs in all clusters, or from DREM analysis. *P*-values are from Fisher’s exact test. Number in blue denotes number of genes in universe not counted inside the Venn diagram. Plot shows the output of DREM with the master regulators listed on top of expression profiles of target gene clusters

We first tested whether a differential gene expression analysis would suffice to prioritize the drivers of rapamycin response. We used LIMMA (Linear Models for Microarray Data) [11] to assess differential expression between the 0 minute and 50 minute time points and selected the top 20 % genes with the most significant *p*-values under the assumption that genes which change their expression are likely to include those driving the phenotypic response. To visualize the data, we took the profiles of the top 20 % genes across all the time points and standardized them on a gene-by-gene basis by subtracting the average expression for that gene and dividing by the standard deviation. We then applied model-based clustering (using the R package *mclust*) to cluster the standardized profiles. We found 25 clusters which showed distinct patterns of expression over time (Fig. 2a) [26]. We carried out functional enrichment analysis by using the R package *GOstats* to apply Fisher’s exact test for overlap with Gene Ontology (GO) Biological Process terms. For each cluster, we computed the *p*-values for all the GO terms, and then adjusted for multiple testing using the Benjamini-Hochberg (BH) method. The results show that many of these clusters represent expected downstream effects of the TOR pathway, including changes in nitrogen metabolism, actin polymerization, and autophagy, as well as a large module of RNA processing genes. We include the full list of enriched GO terms in Additional file 2.

Among the differentially expressed genes, we found no evidence of enrichment in driver genes (Fig. 2b). In fact, the top 10 % of differentially expressed genes was significantly depleted in drivers (*P* = 0.002; Fisher’s exact test). The lack of enrichment was independent of the arbitrary cutoff at 10 %. Traversing down the full list of 5655 genes on the array, ranked according to their LIMMA *p*-values, never resulted in any statistical significance. Finally, the receiver-operator characteristic (ROC) curve shows that driver genes are not overall enriched among the ranking of differentially expressed genes (*P* = 0.58; Kolmogorov-Smirnov [KS]). These results are consistent with previous reports that the genes with the greatest change in mRNA expression are not the genes that drive phenotypic transitions [1].

### Transcription factor enrichment analysis improves detection of drivers of rapamycin response

A common method for finding drivers of gene expression is to look for transcription factor binding sites in the promoters of the genes. We used a high-probability map of TF binding motif sequences [27] to annotate the promoters of the cluster genes. We selected binding sites that passed the *P* < 0.005 cutoff and did not apply any filter based on evolutionary conservation. This produced a network with 118 transcription factors linked to 3942 target genes. To identify enriched TFs, we used Fisher’s exact test to determine the *p*-value for each TF in each cluster. For each cluster, the vector of *p*-values corresponding to all 118 TFs was adjusted using the Benjamini-Hochberg method. Eleven transcription factors were significantly enriched (*P*_*BH*_ < 0.05) in at least one cluster. Ten TFs are drivers of rapamycin response, as defined by the deletion screen. There are only three TFs that overlap between the 11 enriched TFs and the 10 driver TFs, and the overlap is on the border of being statistically significant (*P* = 0.051; Fisher’s exact test) (Fig. 2c). Therefore this method can help detect a small subset of driver TFs.

We also used DREM [13], a method which models bifurcations in time-series expression data and uses TF-target binding sites to extract the regulators responsible for those bifurcations. We ran DREM with default parameters and used as input the same binding site data (see above) and gene expression data (the 30 most sensitive strains at all six time points) as we used for the TF enrichment analysis. DREM identified 15 putative master regulators, three of which are drivers (Fig. 2c). This overlap was not statistically significant (*P* = 0.12; Fisher’s exact test).

### Drivers have high degree in regulatory networks but are not the biggest hubs

We used two network inference methods to infer transcriptional networks from the rapamycin gene expression data (see Methods for further details about network inference). The first, Global Mutual Information Test (GMIT), computes conditional mutual information to find the optimal set of regulators for each gene [28]. We restricted the set of potential regulators to those that have high-probability binding sites in the promoters of the gene. The resulting network had 3615 edges between 109 TFs and 2741 genes. The second, PANDA, uses message-passing to develop an optimal model for the activity of each transcription factor and each target gene’s promoter [17]. The network found by PANDA had 5171 edges between 36 TFs and 3315 genes.

**Fig. 3:**
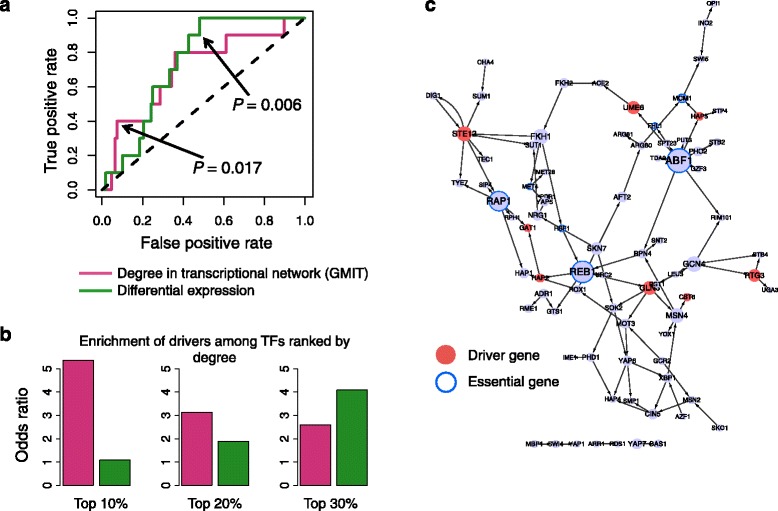
Driver TFs have high degree in inferred regulatory network associated with rapamycin response. **a** Receiver-operator characteristic (ROC) curves showing performance of two different measures – degree in GMIT transcriptional network and differential expression after addition of rapamycin for 50 minutes – in identifying driver TFs. *P*-values are computed using Wilcoxon test. **b** Bar graphs show the odds ratio for the overlap between driver TFs and the top 10, 20 and 30 % of TFs ranked by degree or differential expression. **c** Transcriptional network inferred by GMIT in rapamycin-perturbed yeast. Only TFs and their interactions are shown. Red nodes denote rapamycin driver genes. The size of the node is proportional to its degree in the full network, including all target genes

We tested whether driver TFs are associated with high degree, defined as the number of neighboring nodes on both outgoing and ingoing edges, in the transcriptional network. We ranked the nodes of the GMIT network by their degree and plotted the ROC curve, showing that driver genes tend to have higher degree than expected by chance in transcriptional networks (Fig. 3a). The highest-degree driver TFs are listed in Additional file 3. Since TFs that have bigger changes in expression might have stronger correlations and thus more connections in the network, we tested whether differential expression alone could recapitulate this result. Overall, both differential expression and network degree were significantly enriched in driver TFs (*P* = 0.006 and *P* = 0.017 respectively; Wilcoxon rank-sum test). However, ranking TFs by their network degree yielded a higher odds ratio (OR) at the top 10−20 % of the list than ranking by differential expression (Fig. 3b). This improvement in specificity among the top candidate master regulators would allow more efficient design of further experimental and functional validation.

We repeated the same analysis for the PANDA network (see Additional file 1). The PANDA network had different topology and characteristics than the GMIT network (Fig. 3c and Additional file 1: Figure S2C), due to their very different underlying methodologies. Nevertheless, we found that ranking the TFs by the degree in the PANDA network led to significant enrichment in drivers (*P* = 0.048; Wilcoxon) (Additional file 1: Figure S2A). The top of the list ranked according to degree showed a modest improvement in odds ratio when compared with the list ranked by differential expression (Additional file 1: Figure S2B). These results together show that the number of regulatory interactions is a useful metric for ranking master regulators, and that this is not an artifact of the particular algorithm used to infer them.

Since essential genes tend to have high degree in protein interaction networks, we wondered if the high degree of the driver genes was simply due to them also being essential genes [29]. However, inspection of the inferred transcriptional networks shows that the set of essential TFs does not overlap with the rapamycin driver TFs (Fig. 3c and Additional file 1: Figure S2C). In fact, the highest-degree nodes in the transcriptional network tend to be essential genes. In contrast, the rapamycin response drivers tend to be medium-degree nodes in the transcriptional network.

### Combining protein interaction and transcriptional networks helps predict drivers

**Fig. 4:**
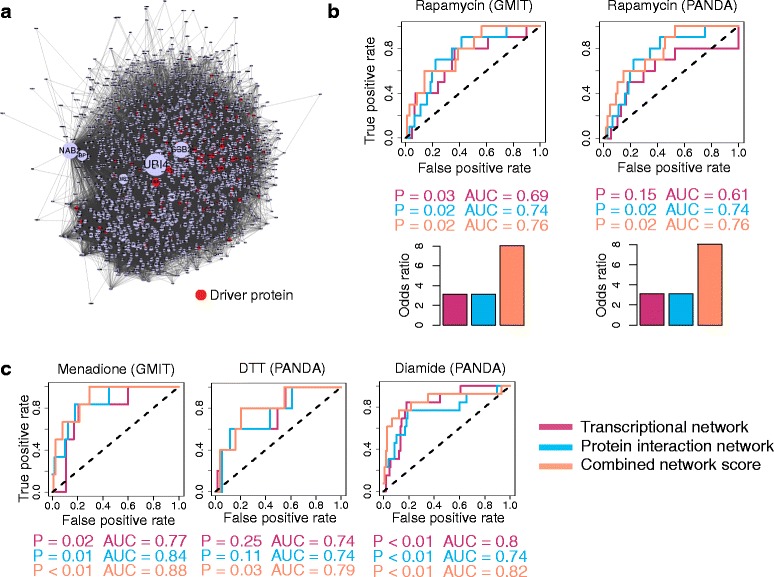
Combining degree in transcriptional network and protein interaction network improves enrichment in driver genes. **a** Protein interaction network. Only the top 2000 most differentially expressed proteins from the rapamycin gene expression data are shown in order to reduce the complexity of the network. Red nodes denote rapamycin driver genes. The size of the node is proportional to its degree in the pictured network. **b** ROC curves showing performance of three different measures – degree in transcriptional network, degree in protein interaction network, and combined network score – in identifying driver TFs. *P*-values are computed using Kolmogorov-Smirnov test. AUC = area under the curve. Bar graphs show odds ratio for the overlap between driver TFs and the top 20 % of TFs ranked by each of the three measures. **c** ROC curves for three other growth conditions, with transcriptional networks inferred using GMIT or PANDA, as indicated

We built a protein interaction network by combining yeast two-hybrid (Y2H) data, reported protein co-complexes, and the list of interactions in the BioGRID (Biological General Repository for Interaction Datasets) database [30–32]. This network had a total of 236577 unique edges between 6466 proteins. Inspecting the network, we found that ranking the nodes by degree resulted in enrichment of rapamycin driver genes (Fig. 4a and b). The highest-degree drivers are listed in Additional file 3. These drivers are not transcription factors but are proteins involved in cell proliferation (SLT2, BRE1) and endocytosis (ACT1, GET2, UBP3).

We hypothesized that the protein interaction network provides information complementary to the transcriptional network, and so the two data sources could be combined for maximal impact. We therefore created a score that combines the degree of each TF in the two networks. The degree distributions of inferred regulatory networks and protein interaction networks are very different, so simply summing the degrees in the two networks can lead to biases. We instead used the rank of the degree of the TF among all other TFs in the network, effectively “normalizing” the two degree distributions so that their characteristics can be combined. Any TF that did not appear in the network (and so had degree 0) was assigned a random rank at the bottom of the list. For every TF, we computed the rank in the transcriptional network *R*_*T*_ and the rank in the protein interaction network *R*_*P*_ and took the average to get a score for that TF: *S = (R*_*T*_ *+ R*_*P*_*)/2*. We then re-ranked the transcription factors according to this new combined score *S*.

The resulting list was highly enriched in driver genes (*P* = 0.02; Kolmogorov-Smirnov) and the top 20 % of the list contained 6 of the 10 driver TFs (Fig. 4b). The combined rank performs better than either the protein interaction or transcriptional degree alone, as measured by several metrics. The area under curve (AUC) statistic for the ROC curve was higher for the combined score (AUC = 0.76) than for the transcriptional network degree (AUC = 0.69) or the protein interaction degree (AUC = 0.74). The overlap of the top 20 % of the TFs as ranked by the combined score was more significant by Fisher’s exact test (*P* = 0.004) and had greater odds ratio (OR = 8.0) than the corresponding overlaps for the transcriptional (*P* = 0.1; OR = 3.1) or protein interaction network (*P* = 0.1; OR = 3.1). The KS statistic was comparable for all three ranked lists (*P* = 0.03 (transcriptional), *P* = 0.02 (protein), *P* = 0.02 (combined)). Therefore, combining the degrees from different network types can help to prioritize true phenotypic drivers, screening out the more general interactors that may appear as dominant hubs in one of the individual networks but do not specifically drive rapamycin response.

We repeated this analysis with the PANDA network and found similar results (Fig. 4b), showing that the combined score is beneficial for any effective network inference algorithm. To ensure that the results were not biased by individual proteomic studies, we also repeated the analysis using only the Y2H protein interaction network [30], which provides an unbiased genome-wide dataset. The conclusion remained the same (Additional file 1: Figure S3). We also tried combining the rank of the protein interaction network degree with the differential expression rank, instead of with the transcriptional network degree. We found that incorporating the protein interactor information resulted in a significant improvement in enrichment among the top differentially expressed TFs (see Additional file 1: Figure S4 and Supplementary Text in Additional file 1 for further details).

We next examined the degree of driver TFs in conditions other than rapamycin. We downloaded gene expression data measured using custom DNA microarrays in the study by Gasch et al. (http://genome-www.stanford.edu/yeast_stress/data/rawdata/complete_dataset.txt) [33]. We identified all the conditions that had more than five microarray samples (either replicates or time course) and had been interrogated for drivers using a deletion library screen. These six conditions were: heat shock, hydrogen peroxide, menadione, DTT, sorbitol, and diamide (see Methods for more details on data preprocessing). We collected a set of driver TFs for each condition from the following studies: heat shock [4, 5], hydrogen peroxide and menadione [34], DTT [35], sorbitol [1] and diamide [6]. For each condition, we inferred transcriptional networks using both GMIT and PANDA. We then ranked all TFs by their degree in either the transcriptional or the protein interaction networks or by the combined score *S* (Fig. 4c and Additional file 1: Figure S5 and S6). Many of the inferred transcriptional networks were not significantly enriched in driver TFs. The gene expression datasets had only about ten samples each, so the quality of the inferred networks may have decreased due to the low sampling rate. Only three of the six conditions—menadione, DTT and diamide—showed good enrichment for drivers among the high degree TFs, with an AUC greater than 0.7 (Fig. 4c).

When the TFs were ranked by their degree in the protein interaction network, four of the six stress conditions showed significant enrichment in drivers: hydrogen peroxide and diamide (*P* < 0.01), heat shock (*P* = 0.04) and menadione (*P* = 0.01). Furthermore, in the cases where the transcriptional network showed enrichment in drivers with AUC > 0.7, we found that ranking the TFs by the combined score led to improved AUC and *p*-values (Fig. 4c). For example, when the menadione GMIT network was combined with the protein interaction network, the AUC increased from 0.77 (transcriptional only) or 0.84 (protein interaction only) to 0.87 and the *p*-value decreased correspondingly. The results for the networks with AUC < 0.7 are shown in Additional file 1: Figures S5 and S6 for completeness.

In summary, integrating protein interaction data with the transcriptional network almost always increased the power to rank drivers, as compared to the transcriptional network alone. Secondly, in the cases where the transcriptional network was itself enriched in drivers, combining the networks using our degree-rank score improved enrichment in drivers beyond either the protein interaction network or transcriptional network alone.

### High degree nodes in viral oncogene transcriptional network are enriched for cancer drivers

**Fig. 5:**
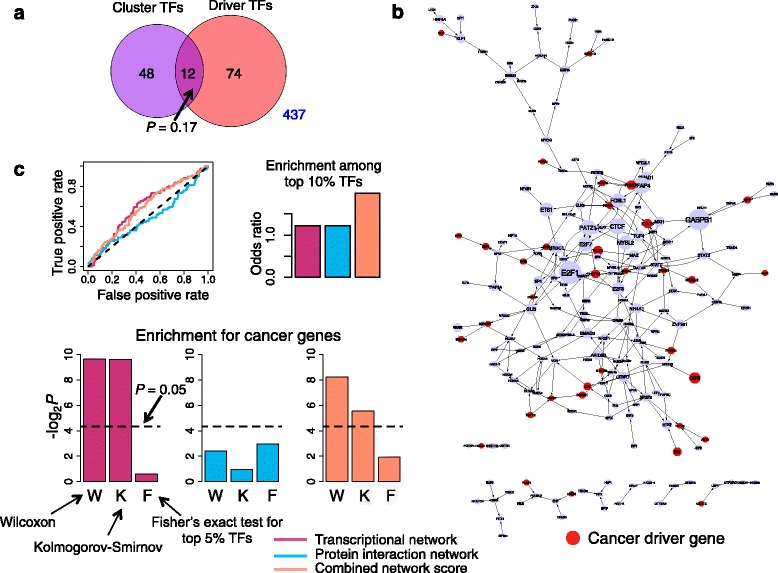
Combined score for viral oncogene-associated GMIT network and protein interaction network improves specificity for identifying cancer genes. **a** Venn diagram depicting overlap between enriched TFs in gene expression clusters and cancer driver TFs. *P*-value computed using Fisher’s exact test. Number in blue denotes number of genes in universe not counted inside the Venn diagram. **b** GMIT transcriptional network for the transforming viral oncogenes. Sanger cancer genes are depicted as red nodes. Size of node is proportional to degree of node. **c** Line plot shows the ROC curves for overlap with cancer drivers for each of three network measures – degree in GMIT network, degree in yeast two-hybrid network, or combined network score. Bar graph (above) shows the odds ratio of the overlap between cancer drivers and the top 10 % (or top 57 out of 571) TFs. Bar graphs (below) show the significance of the enrichment in cancer drivers using three different statistical tests: W = Wilcoxon rank test, K = Kolmogorov-Smirnov test, F = Fisher’s exact test for the overlap between the top 5 % (or top 29 out of 571) ranked TFs and cancer drivers. Black dotted line indicates lower bound for statistical significance

We constructed a transcriptional network for a human disease-associated dataset (GEO Accession: GSE38467) [36]. These data were generated by introducing 63 proteins from tumor viruses individually into IMR-90 normal human fibroblasts. The resulting cell lines were assayed for gene expression using Affymetrix Gene 1.0 ST microarrays, and host interactors of the viral proteins were identified by both tandem affinity purification followed by mass spectrometry (TAP-MS) and by Y2H. The interactors of viral proteins were found to be enriched in cancer drivers [36]. The top 15 % (2944 genes) most frequently differentially expressed genes across all viral perturbations were grouped using model-based clustering into 31 clusters. DNase-I-hypersensitivity data for IMR-90 cells was integrated with TF binding sites to build an IMR-90-specific prior network consisting of 571 transcription factors and 4059 target genes [36]. Sixty TFs were categorized as actively regulating the clusters, because they were differentially expressed in response to viral proteins, and had target genes that were significantly enriched in at least one cluster [36]. However, these TFs were not significantly enriched in cancer drivers, as defined by the list of known cancer genes from the Sanger Cancer Gene Census (*P* = 0.17; Fisher’s exact test) (Fig. 5a) [37].

**Fig. 6:**
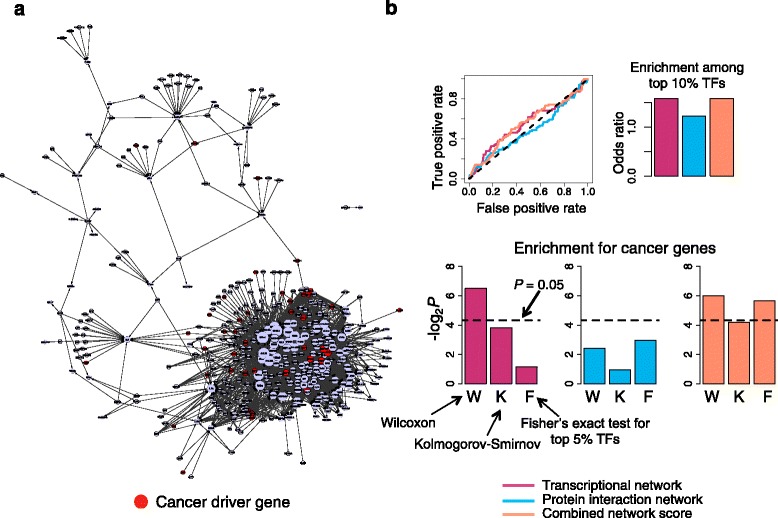
Combined score for viral oncogene-associated PANDA network and protein interaction network improves enrichment and specificity for identifying cancer genes. **a** PANDA transcriptional network for the transforming viral oncogenes. Sanger cancer genes are depicted as red nodes. Size of node is proportional to degree of node. **b** Line plot shows the ROC curves for overlap with cancer drivers for each of three network measures – degree in PANDA network, degree in yeast two-hybrid network, or combined network score. Bar graph (above) shows the odds ratio of the overlap between cancer drivers and the top 10 % (or top 57 out of 571) TFs. Bar graphs (below) show the significance of the enrichment in cancer drivers using three different statistical tests: W = Wilcoxon rank test, K = Kolmogorov-Smirnov test, F = Fisher’s exact test for the overlap between the top 5 % (or top 29 out of 571) ranked TFs and cancer drivers. Black dotted line indicates lower bound for statistical significance

We applied PANDA and GMIT to the gene expression data to construct viral oncogene-associated transcriptional networks. We first created two groups of samples, one corresponding to the 37 viral proteins that are classified as “transforming” due to their tumorigenic properties, and the second corresponding to all control cell lines (including IMR-90 cells expressing empty vectors or green fluorescent protein (GFP)) that were part of the gene expression dataset. For the GMIT analysis, we computed conditional mutual information between each gene and the regulators whose DNase-I-hypersensitive binding sites are present in the promoter of the gene (Fig. 5b). For the PANDA analysis, we combined the microarray data for each sample group with the IMR-90-specific prior network described above to infer edge weights for each sample group. The two sets of edge weights were then compared (see Methods for further details) to find the significantly active edges in the viral oncogene-associated regulatory network (Fig. 6a). We ranked the transcription factors by their degree in the two networks and evaluated the rankings for enrichment in cancer genes from the Sanger Cancer Gene Census. Of the 571 known cancer genes, 86 of them are transcription factors with known binding motifs. In both the GMIT and PANDA networks, we found that TFs ranked by degree were significantly enriched in these driver TFs (*P* = 0.001 and *P* = 0.011 respectively; Wilcoxon) (Fig. 5c and 6b). On the other hand, we found a lack of enrichment among the top 5 % TFs by degree, consistent with our observation that the highest-degree nodes in regulatory networks do not tend to be phenotypic drivers (*P* = 0.66 (GMIT) and *P* = 0.45 (PANDA); Fisher’s exact test).

We compared this with simply ranking the transcription factors by differential expression between the “transforming” viral gene samples and the control samples. We ranked the TFs by their *p*-value according to LIMMA. The top 5 % was significantly enriched in cancer drivers (*P* = 0.02; Fisher’s exact test) but the ranking as a whole was not significant (*P* = 0.44; Wilcoxon). Furthermore, the top-ranked drivers from differential expression analysis had little overlap with the top-ranked drivers from network analysis (Additional file 4). Therefore, the network approach can be used to prioritize new drivers that cannot be found using standard analysis of gene expression data.

### Integrating protein interactions improves enrichment of cancer genes

We used the human Y2H dataset to build a protein interaction network (http://interactome.dfci.harvard.edu/H_sapiens/download/HI-II-14.tsv) [38]. This dataset corresponds to ~13,000 proteins and covers about 42 % of the search space of potential protein-protein interactions. We did not use literature-based protein interaction databases because published studies on human protein interactions are heavily biased towards known cancer genes, so the degree of cancer drivers in such a network cannot be interpreted in a meaningful manner.

We ranked each TF by its degree in the Y2H network and computed the enrichment in Sanger cancer drivers using several different statistical tests (Fig. 5c). The top 5 % TFs by Y2H network degree did not demonstrate significant enrichment in cancer drivers (*P* = 0.13; Fisher’s exact test) and the overall ranking was also not significant (*P* = 0.19, Wilcoxon; *P* = 0.52, Kolmogorov-Smirnov). We next computed the combined score that we defined earlier as the average of the degree ranks in the transcriptional and protein interaction networks: *S = (R*_*T*_ *+ R*_*P*_*)/2.* For the GMIT network, the combined ranking did not surpass the protein interaction network degree in the enrichment of drivers among the top 5 % TFs (*P* = 0.26 [combined], *P* = 0.66 [GMIT only], *P* = 0.13 [Y2H only]; Fisher’s exact test) (Fig. 5c). On the other hand, the combined score performed better (OR = 2) than either individual network (OR = 1.2) when considering the top 10 % of TFs. The overall significance of the combined ranking (*P* = 0.003; Wilcoxon) was similar to that of the transcriptional network degree (*P* = 0.001), and was an improvement over the PPI network degree (*P* = 0.19). In summary, the combined network metric did not improve the overall ranking, but did improve the specificity among the top 10 % of the ranked list.

In the case of the PANDA network, ranking the TFs using this combined score led to marked improvement in the enrichment in cancer genes, both among the top 5 % of the list (*P* = 0.02, Fisher’s exact test) as well as throughout the entire ranking (*P* = 0.016, Wilcoxon test; *P* = 0.054, Kolmogorov-Smirnov) (Fig. 6b). The particular characteristics of the PANDA algorithm could explain why the combined score works better with the PANDA network than with the GMIT network. The improved enrichment from combining the networks depends on the existence of drivers that are moderately active in both the regulatory and protein interaction networks, and which then score higher than the hubs in each individual network. Since PANDA independently models the activity of TFs and target promoters, it can better detect TFs that are post-translationally regulated and whose expression doesn’t necessarily have high mutual information with its targets’ expression. Mammalian regulation relies more on such post-translational and epigenetic mechanisms – like phosphorylation, acetylation, chromatin modifications and enhancer regions – than simpler organisms like yeast. Methods like PANDA could be better at teasing out these complex patterns of regulation in human cells, and thus contribute more to the combined network metric.

As in the case of the rapamycin data, we compared these results with directly combining the differential expression rank and the degree in the protein interaction network. In contrast to the rapamycin case, we found that using differential expression to compute the combined metric resulted in decreased enrichment both in the top 10 % of the list as well as throughout the ranking (Additional file 1: Figure S7). See Supplementary Text in Additional file 1 for more details.

We examined in greater detail the top 30 regulators ranked by the combined network score applied to the PANDA network, and found that 10 are validated cancer genes included in the Sanger Cancer Gene Census (Table 1). In addition, 5 more regulators have been functionally linked to cancer in multiple reports. The regulator ranked third is TFCP2, which is known to be an oncogene in hepatocellular carcinoma (HCC), and enhances angiogenesis and chemoresistance in several cancer types [39, 40]. The regulator ranked eighth, SMAD3, is a well-known signal for growth and development and plays a role in carcinogenesis and metastasis [41–43]. The ninth TF is BCL6B, a protein that associates with BCL6, an established clinical marker of lymphoma [44]. BCL6B is thought to be a tumor suppressor in colorectal cancer and HCC [45, 46]. MXI1 antagonizes c-MYC and appears to have tumor suppressive capacity in several cancers, including neuroblastoma and renal carcinoma [47, 48]. Finally, the TF ranked #28, TFAP4, is a target of c-MYC and is involved in promoting cell cycle progression [49]. It has been linked to non-small cell lung cancer and colorectal cancer [50–52]. All told, 15 (or 50 %) of the top 30 TFs have strong evidence in the literature for being master regulators in cancer.

**Table 1:** Top thirty viral oncogene-associated TFs ranked by combined network score

Rank	Regulator	PPI degree	Transcr. degree	Combined score *S*	Annotation
1	TCF4	132	41	8.5	
2	TCF12	29	43	11	Sanger cancer gene
3	TFCP2	47	32	16.5	Oncogene in hepatocellular carcinoma (HCC); enhances angiogenesis and chemoresistance [39, 40]
4	ZBTB7B	12	43	17.5	
5	SP4	11	46	17.75	
6	IKZF1	57	28	19.25	Sanger cancer gene
7	MYOG	11	42	21.5	
8	SMAD3	8	34	31.5	Plays role in carcinogenesis and metastasis [41–43]
9	BCL6B	14	20	32.75	Tumor suppressor in colorectal cancer and HCC [45, 46]
10	WT1	3	43	39.5	Sanger cancer gene
11	LMO2	66	12	40.25	Sanger cancer gene
12	SP2	3	36	45.5	
13	CREB5	35	11	48.25	
14	TCF3	2	51	48.5	Sanger cancer gene
15	MXI1	11	13	49.5	Potential tumor suppressor in neuroblastoma and renal cancer [47, 48]
16	NRF1	4	20	50.5	
17	CREB3L1	16	11	52	Sanger cancer gene
18	USF1	5	15	54.5	
19	GABPB1	10	12	56	
20	NHLH1	2	38	56.25	
21	MAX	3	20	57.25	Sanger cancer gene
22	NR1H3	3	17	61.75	
23	USF2	3	15	65	
24	SMAD4	2	20	69.25	Sanger cancer gene
25	STRA13	2	20	69.25	
26	NR1H2	2	18	72	
27	EBF1	2	15	77	Sanger cancer gene
28	TFAP4	3	9	82.5	Linked to non-small cell lung cancer and colorectal cancer [50–52]
29	RXRB	2	12	85.25	
30	NFE2L2	2	12	85.25	Sanger cancer gene

## Conclusions

Gene expression analysis has been widely used to assay how biological systems respond to a variety of perturbations, including environmental stresses and drug treatments. In particular, gene expression profiling is used to characterize tissue samples from patients with diseases of complex origin. These profiles are analyzed to identify genes perturbed by the disease and to potentially discover new biomarkers. However, genome-wide screens and expression profiling in yeast have established that the most differentially expressed genes are not the same genes that drive the response to a perturbation. Therefore, it remains a challenge to prioritize drivers of complex phenotypes, such as disease and drug response, using gene expression data.

Most genes do not work in isolation but rather function together in pathways and complexes. They form a network of molecular interactions whose activity and dynamics determine the biological processes that are deployed in response to changes in the environment. Thus, a natural alternative to studying differential gene expression is to examine the underlying gene interaction networks and their structure to extract features that drive phenotypic transitions. Analysis of protein-protein interaction networks has found that highly connected “hub” nodes are significantly enriched in the essential genes that are necessary for survival. We found that the regulators that drive the response to rapamycin or other yeast stress conditions also have higher degree in the PPI network than expected by chance. However, the PPI network is measured under normal growth conditions and thus represents a context-independent pool of all possible interactions between proteins. Master regulators, on the other hand, are highly dependent on the context and the exact perturbation under study. Therefore, more data is necessary to determine which high-degree proteins are important for mediating the cellular response to any particular condition.

We addressed this challenge by using gene expression data to build context-specific regulatory networks. To test the generality of our approach, we used two different algorithms to integrate gene expression data with transcription factor binding sites in order to infer realistic models of regulation. We used GMIT to compute conditional mutual information between a target gene and the potential regulators that have binding sites in the promoter of that gene, and PANDA to apply message-passing to find the best-fit model for TF and target gene activity, and their interactions. We hypothesized that the regulators that drive response to environmental perturbations would have high degree in this network. Applying this analysis to rapamycin response in yeast, we observed that driver TFs indeed have higher degree than expected by chance. However, the highest-degree nodes are not drivers but instead tend to be either essential for normal growth or widely active regulators that partner with RNA polymerases and are involved in housekeeping processes. This suggests that when a cell is perturbed, it activates specific functional pathways in tandem with more general activators or repressors that modulate the landscape of transcription. The former are associated with the response to that particular perturbation, whereas the latter lead to sweeping patterns of expression that dominate differential expression patterns. As a corollary, though the true drivers have significantly higher degree on average, the degree alone is not enough to enrich for the true phenotypic drivers in regulatory networks with high specificity.

We next asked whether combining transcriptional networks with the protein-protein interaction data could improve the detection of drivers. We reasoned that the two data types could complement each other, because protein interactions and gene regulation are inherently distinct processes but both significantly contribute to the functioning of the cell. We first noted that the degree distributions of the two networks are very different. The regulatory networks we inferred were generally smaller and had less of the “heavy tail” characteristic of scale-free networks. In other words, they did not have as many nodes with extremely high degree. The protein interaction networks were larger and had more high-degree hubs. Instead of directly combining the degrees in the two networks, we first ranked the nodes by their degree in each network separately, and then computed the average of the ranks. Similar to nonparametric statistical tests like the Wilcoxon rank-sum test, this procedure ensures that the score is not dependent on the exact forms of the degree distributions.

We applied this combined PPI and transcriptional network score to compute the enrichment in drivers in the response of yeast to rapamycin. The combined score was able to better prioritize drivers, especially at the top of the list of ranked TFs. This was because the regulators that control specific functions downstream of the TOR pathway had more protein interactions than the hub TFs that activate general stress response or other basic cellular programs. We next applied the combined network score to other yeast conditions. In the cases where the regulatory network alone was enriched in drivers (menadione, DTT and diamide), we again found that combining it with the PPI data improved our ability to prioritize driver TFs.

We wondered whether this principle could be applied to human biology. We used gene expression profiles derived from primary human fibroblasts expressing a variety of viral oncogenes, and applied both GMIT and PANDA to build viral oncogene-associated regulatory networks. We found that established cancer master regulators, as annotated in the Sanger Cancer Gene Census, tended to have higher degree in this network than expected by chance. However, the highest-degree nodes in the network comprised a cancer gene “desert” and were not enriched in drivers. We then integrated the regulatory networks with the human yeast two-hybrid interactome using our combined network score. In the case of the GMIT network, integrating it with the Y2H degree increased enrichment among the top 10 % of the TFs, whereas for the PANDA network, the enrichment was increased among the top 5 % of the TFs and the significance of the overall TF ranking increased as well. In particular, the combined PANDA and Y2H network score boosted the number of cancer genes at the top of the ranked list of regulators. Six of the ten most highly ranked regulators have strong evidence in the literature of causal links to carcinogenesis, angiogenesis, and metastasis.

In the case of rapamycin in yeast, we found that combining differential expression with protein interactions could produce the same level of enrichment in drivers as using the transcriptional network. In contrast, in the human dataset, the combination of differential expression and PPI degree was not as effective as the combined network score. This could be due to differences in the complexity of regulatory networks in yeast and humans. A multilayered regulatory system, like that of humans, could result in more complicated TF activity patterns that require analysis beyond simply comparing mRNA expression levels. The network metric may therefore be more widely applicable as it captures the effects of genes that do not change in expression but serve as links between genes that do.

Overall, we found the best enrichment and specificity for drivers by using a combined network score that prioritizes TFs that regulate many target genes and also physically interact with many proteins. One simple reason for this may be that integrating the two data types helps to buffer against the noise and variation in each network. The most robust signal remains after being filtered through the two independent datasets. However, another possible interpretation is that the high degree in the protein interaction network represents more activity in the signaling pathways of the cell, and high degree in the transcriptional network means a TF has more gene targets and can affect functional processes through the regulation of gene modules. The drivers ranked higher by the combined network metric may tend to significantly interact with both protein and mRNA regulation in the cell.

**Fig. 7:**
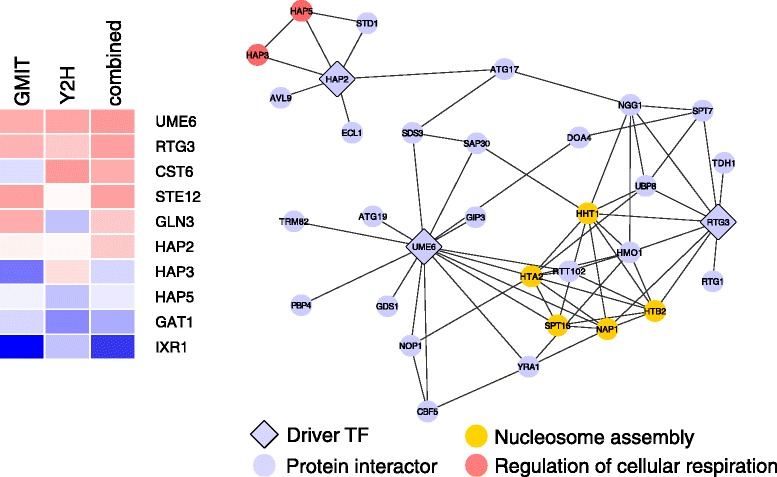
Rapamycin drivers with high combined network score interact with proteins enriched for nucleosome assembly and respiration. Heatmap shows the ranks of all rapamycin driver TFs according to either the transcriptional network degree, the protein interaction degree, or the combined network score, with red depicting higher ranks and blue depicting lower ranks. Network diagram shows all direct protein interactors of the rapamycin driver TFs that had a higher rank in the combined network score than in either individual network alone. Edges represent evidence of direct protein interaction from yeast two-hybrid experiments

**Fig. 8:**
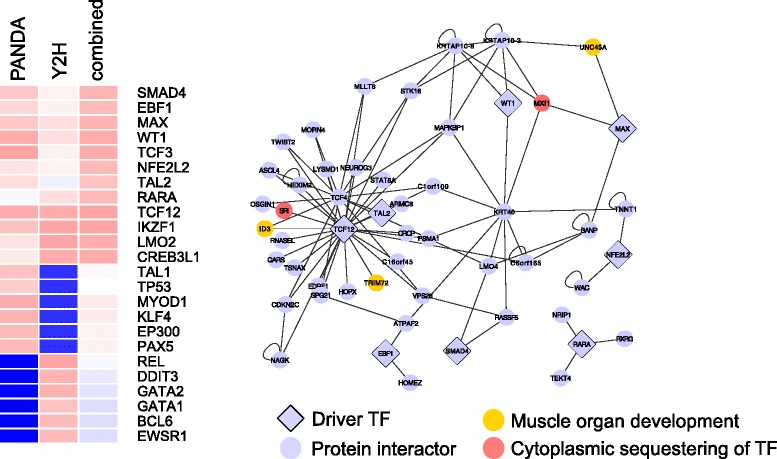
Cancer drivers with high combined network score interact with proteins enriched in cellular localization or developmental pathways. Heatmap shows the ranks of cancer drivers that were among the top 10 % TFs according to either the transcriptional network degree, the protein interaction degree, or the combined network score, with red depicting higher ranks and blue depicting lower ranks. Network diagram shows all direct protein interactors of the cancer driver TFs that had a higher rank in the combined network score than in either individual network alone. Edges represent evidence of direct protein interaction from yeast two-hybrid experiments

We delved deeper into how the protein interaction network data helps to prioritize driver TFs. We first used heatmaps to visualize the ranks of driver TFs in each network individually, and in the combined network score (see Figs. 7 and 8 and Supplementary Text in Additional file 1). Inspecting the heatmaps, we observed that there are two types of driver TFs that end up being highly ranked by the combined network score. The first class of TFs already have a high rank in either the transcriptional or protein interaction network, and therefore are still highly ranked in the combined score. The second class of drivers has a more moderate degree in both the transcriptional and protein interaction networks, but their score in the combined network score is even higher than each individual rank, leading to an overall increase in the specificity and enrichment of drivers at the top of the list. This second group of drivers represents the power of combining the network degrees.

We examined the protein interactors of this second group of drivers to better understand the biological processes that contribute to the increased enrichment in the combined network score (see Additional files 1 and 5 for more details). We found that many of the interactors are involved in chromatin assembly and histone modification. For example, RSC3 is a member of a chromatin-remodeling complex and interacts with YAP1, a driver of menadione response. The rapamycin driver RTG3 interacts with the histones HHT1, HTA2, and HTB2. Another commonly enriched function involves nuclear transport and TF localization, including CRM1, which exports mRNA and protein from the nucleus and interacts with YAP1, or SRI, a human protein that can bind calcium and translocate to the cytoplasm and regulate the activity of calcium channels. In the human network, we also found a significant enrichment for developmental signaling pathways, exemplified by proteins like ID3 and UNC45A, which are involved in cell differentiation and proliferation, and which interact with the cancer drivers MAX and TCF12 respectively.

Taken together, these observations suggest that the combined network score prioritizes TFs that regulate functional gene modules and are also highly regulated themselves at the protein level, either by nuclear translocation, chromatin modification partners, competitive binding, or signaling pathways. Thus they constitute flexible channels by which condition-dependent signals are transmitted through the protein interaction network to the transcriptional network. This suggests a general principle for biological networks: that the nodes responsible for biological state transitions tend to have the highest rate of information flowing through them, as signals are transmitted from the environment through the molecular networks and to the final response (or phenotype) of the cell. Such an organizing principle could be applied to integrate other types of networks as well, like those constructed using metabolomic or phosphoproteomic data.

This also suggests that therapeutic interventions ought to target highly regulated nodes in disease-associated networks. Transcriptional networks could be constructed from gene expression profiles of patients, and combined with protein interaction databases and other information from the literature to prioritize regulators that are driving the disease, and to identify potential therapeutic strategies that target these pathways. We believe that further development of principled techniques for network integration and analysis would be beneficial for interpreting biomedical data and finding the elements that drive biological states and diseases.

## Methods

### Data preprocessing

To preprocess the data from [33], all rows and columns that had more than 80 % missing values were eliminated from the analysis. The rest of the missing values were imputed using the k-nearest-neighbor algorithm. The corresponding driver genes were found in the following studies: heat shock [4, 5], hydrogen peroxide and menadione [34], DTT [35], sorbitol [1] and diamide [6]. For hydrogen peroxide and menadione, we used a cutoff of 1.5 for the ratio of growth under normal conditions to growth under stress conditions, as recommended by the authors [34]. For the sorbitol screen, we used a significance cutoff of 20 for the generation 5 experiments, and a significance cutoff of 100 for the generation 15 experiments, also as recommended by the authors [1]. Note that, in all cases, we included genes associated with both resistance and sensitivity whenever possible.

### Yeast annotation

Wherever yeast genes were annotated as gene symbols rather than systematic open reading frames (ORFs), we translated all annotations to systematic ORF names using a table of chromosomal features downloaded from the Saccharomyces Genome Database (http://downloads.yeastgenome.org/curation/chromosomal_feature/dbxref.tab) on May 5, 2015.

### Inferring transcriptional networks

To bin expression data for GMIT network inference, we computed quantiles for each gene and classified its expression in each sample into one of three bins. Since it is computationally intensive to exhaustively search every combination of regulators, we set the number of potential regulators for each gene to ten. For promoters that contained more than ten unique binding sites, we ranked each potential regulator by the absolute value of its Pearson correlation with the target gene. We then used the top ten TFs to search exhaustively for the maximum conditional mutual information using the GMIT method as implemented in C++. For the rapamycin and viral oncogene data, we used a stringent *p*-value cutoff of 0.001; for the rest of the yeast stress conditions, we used a *p*-value cutoff of 0.05 due to the smaller number of samples. Note that, although GMIT can be applied to learn dynamic networks by correlating time-lagged expression profiles, in this case, we used it to learn a static network and did not incorporate a time lag. Since there was no dynamic component to the inferred network, we made sure to not allow self-loops, as every TF would trivially have maximal correlation with its own expression.

We ran PANDA with default parameters and no protein-protein interaction data. For the yeast data, we ran PANDA on both the original gene expression data as well as a randomized version. The randomization was carried out by independently permuting both the gene and sample identities. To create the final network, we converted the z-scores output by PANDA for each edge to a probability, assuming a normal distribution. For each edge, we then computed the difference in probability between the original and randomized data. This value was then multiplied by the probability of the edge being present in the original data. The final edge score thus evaluates whether a particular edge is significantly present in the experimental condition, and is also significant compared to randomized data [53]. We constructed the final network using all edges with a score above 0.8, as described in [53]. For the viral oncogene data, we used the control samples as a comparison, instead of creating an artificially randomized dataset.

### Code

All codes and data input files that were used to arrive at the above results can be found at https://bitbucket.org/meghapadi/networkdriverdiscovery/.

## Availability of supporting data

The datasets supporting the analysis in this article are available from the following repositories and publications.

Yeast rapamycin gene expression: http://www.ebi.ac.uk/arrayexpress/experiments/E-MTAB-412/

Yeast drug-perturbed growth rates: [24]

Yeast rapamycin driver genes: [25]

Yeast ORF annotation: http://downloads.yeastgenome.org/curation/chromosomal_feature/dbxref.tab

Yeast TF binding sites:

http://fraenkel.mit.edu/improved_map/orfs_by_factor.tar.gz

Yeast essential genes:

http://www-sequence.stanford.edu/group/yeast_deletion_project/Essential_ORFs.txt

Yeast Y2H data: [31] and http://interactome.dfci.harvard.edu/S_cerevisiae/download/Y2H_union.txt

Yeast BioGRID data: http://thebiogrid.org/download.php (accessed on May 18, 2015)

Yeast stress gene expression: http://genome-www.stanford.edu/yeast_stress/data/rawdata/complete_dataset.txt

Yeast heat shock driver genes: [4] and [5]

Yeast hydrogen peroxide and menadione driver genes: http://depts.washington.edu/sfields/deletion/index.html

Yeast DTT driver genes: [35]

Yeast sorbitol driver genes: http://genomics.lbl.gov/YeastFitnessData/websitefiles/cel_index.html

Yeast diamide driver genes: [6]

Human viral gene expression: http://www.ncbi.nlm.nih.gov/geo/query/acc.cgi?acc=GSE38467

Sanger cancer gene census: http://cancer.sanger.ac.uk/census/

Human Y2H data: http://interactome.dfci.harvard.edu/H_sapiens/download/HI-II-14.tsv

## Additional files

Additional file 1: Supplementary Text and Figures. Supplementary text describes (a) the robustness of the results to the growth rate cutoff for yeast strains, (b) an analysis combining differential expression with protein interaction degree, and (c) the characterization of the protein interactors of driver TFs. Figures present results for (i) network integration for rapamycin data with alternative growth cutoff, (ii) PANDA analysis of rapamycin expression data, (iii) rapamycin network integration using only yeast two-hybrid data, (iv) results of combining differential expression with PPI degree, (v) ROC curves for network integration in multiple yeast conditions, and (vi) the rank characteristics and local PPI neighborhoods of driver TFs for menadione, DTT and diamide. (PDF 1304 kb) Additional file 2:
**GO term enrichment for expression-based clusters in rapamycin-treated yeast.** (XLSX 161 kb) Additional file 3:
**Top TF drivers of rapamycin response ranked by degree in transcriptional or PPI network.** (XLSX 57 kb) Additional file 4:
**Top transcription factors, as ranked either by 1) differential expression by transforming viral oncogenes, 2) degree in the GMIT network or 3) degree in the PANDA network.** (XLSX 41 kb) Additional file 5:
**Lists of enriched GO terms for the direct protein interactors of subsets of driver TFs that are ranked higher using the combined network score than either individual network.** Lists correspond to the following conditions: rapamycin, diamide and menadione in yeast, and viral oncogene perturbation in humans. (XLSX 77 kb)

## Abbreviations

TF: Transcription factor
DREM: Dynamic Regulatory Events Miner
PANDA: Passing Attributes between Networks for Data Assimilation
PPI: Protein-protein interaction
TOR: Target of rapamycin
DTT: dithiothreitol
RMA: Robust multi-array average
LIMMA: Linear models for microarray data
GO: Gene Ontology
ROC: Receiver-operator characteristic
BH: Benjamini-Hochberg
GMIT: Global Mutual Information Test
BioGRID: Biological General Repository for Interaction Datasets
AUC: Area under curve
KS: Kolmogorov-Smirnov
TAP-MS: Tandem affinity purification followed by mass spectrometry
GFP: Green fluorescent protein
OR: Odds ratio
HCC: Hepatocellular carcinoma
ORF: Open reading frame
