# Supplementary material for: Integrating transcriptional and protein interaction networks to prioritize condition-specific master regulators
Source: BMC Syst Biol. 2015 Nov 14;9:80. doi: 10.1186/s12918-015-0228-1 (PMC4650867; doi:10.1186/s12918-015-0228-1)

Supplemental material for: Integrating transcriptional and protein interaction networks to prioritize condition-specific master regulators

I. Robustness of enrichment analysis to the growth rate cutoff for yeast strains

We selected the 53 strains of yeast that had a lower growth rate in rapamycin than under normal growth conditions. To run GMIT, we proceeded as described in the Methods section, redoing every step for the whole dataset of 53 strains, including data binning, filtering the prior network, and computing conditional mutual information. We also ran PANDA on this expanded dataset, redoing the randomization and the network inference following the identical protocol described in the Methods. As we had done with the smaller 30-strain dataset, we then computed the degree of each transcription factor in the GMIT or PANDA networks, ranked them and combined it with the rank of the degree in either the full protein interaction network or the yeast two-hybrid network. The results, including ROC curves, AUC values, p-values from the Kolmogorov-Smirnov test, and odds ratios for the top 20% TFs ranked by each metric, are shown in Supplementary Figure 1. In every case but one, the enrichment for driver TFs among the top-ranked regulators is highest for the combined metric. The exception is the combination of the PANDA network with the Y2H network, where the odds ratio for the combined metric is the same as the odds ratio for the PANDA network alone; however, even in this case, the p-value and AUC of the overall ranking (P = 0.02, AUC = 0.74) performs better than each network individually (P = 0.1, AUC = 0.6 [PANDA]; P = 0.03, AUC = 0.72 [Y2H]). Therefore, the improvement in enrichment appears to be a general feature of the combined network score, and it is independent of changing the number of samples included in the expression dataset, the type of network inference method, the type of protein interaction network, or even changing all of these factors at the same time.

II. Combining differential expression with protein interaction network

We calculated the differential expression rank R_D of all the transcription factors by ranking the B-statistic computed using *limma*. We then combined it with the PPI rank to compute the score $S_D = (R_D + R_P)/2$. For the rapamycin data, we plotted the ROC curves and odds ratios corresponding to differential expression, degree in transcriptional network (either GMIT or PANDA), degree in the PPI network (either the full network or Y2H only), the combined network metric S, and the combined differential expression and PPI metric S_D. For the viral oncogene data, we followed the same procedure, except with only one type of PPI network (Y2H).

The results for rapamycin are shown in Supplementary Figure 4. In every case, combining the differential expression ranking with the PPI degree resulted in an improvement in the enrichment in driver TFs. The improvement was comparable to the combined network score, as reflected in the ROC curves as well as the odds ratios. One exception was that the odds ratio for the top 20% ranked TFs was higher for the combined network metric in the case of the Y2H protein interaction network. However, in general, the combined differential expression metric performed well in the context of rapamycin response in yeast.

The results for the viral oncogene data are shown in Supplementary Figure 7. The combination of differential expression and protein interaction network did not perform as well as the combined network degree, in the case of both GMIT and PANDA networks, and both in terms of the specificity of the top 10% of the TFs as well as over the entire ranking (as visualized in the ROC curves). One possible reason is that the set of differentially expressed drivers has different characteristics than the set of high degree drivers in the PPI network, so

that combining the two types of information does not result in a higher overall ranking for the drivers. This could be related to the fact that some cancer drivers are post-translationally regulated. This class of drivers may be more likely to have high degree in the transcriptional network rather than be differentially expressed, because their activity does not correlate as well with their mRNA expression as it does in simpler organisms like yeast. Such post-translationally regulated drivers would have better overlap with the class of drivers that have multiple protein interactors. This would result in better enrichment for the combined network score.

III. Characterizing the local network of driver TFs

For each condition, we computed the ranks of the driver TFs with respect to 1) the transcriptional network degree (R_T), 2) the protein interaction degree (R_P), and 3) the combined network score (R_S). We visualized the rankings by making heatmaps of this *TF x score* matrix. In order to improve visualization, we normalized the matrix by subtracting the mean value over the whole matrix from each element. This allows the eye to distinguish high from low ranks by color, while still being able to compare values between different rows and columns. We also hierarchically clustered the rows (TFs) using Euclidean distance and complete linkage. The results are shown in Figures 7 and 8 and Supplementary Figure 8. From the heatmap, it is apparent that there are several types of drivers: the ones that rank highly on the transcriptional network and carry that over to the combined metric, the ones that rank highly on the protein interaction network and carry that over, and finally the ones that have moderate ranks for each network separately, but which are boosted by combining the two networks.

In each condition, we identified this third type of driver TF that had a higher ranking in the combined network score than in both of the individual network scores, i.e. all driver TFs such that (R_S>R_T) AND (R_S>R_P). For these “boosted” TFs, we characterized their neighborhood in the yeast two-hybrid protein interaction network, to get more insight into how data on protein interactions can contribute to prioritizing drivers. We used the Y2H network in order to avoid any literature bias. We pooled all the direct interactors of all the “boosted” TFs in each condition, and tested this set of proteins for GO term enrichment using Fisher’s exact test and Benjamini-Hochberg adjustment. As our universe or null set of genes, we used all the proteins that appear at least once in the Y2H dataset to control for any common characteristics of proteins that are easier to assay by Y2H. We note that the results were qualitatively similar even when using the full coding gene universe. Comprehensive lists of enriched GO terms are presented in Additional File 5. Below, we describe a few examples from each condition.

In the case of rapamycin, the “boosted” driver TFs are HAP2, RTG3, and UME6. They interact with a set of proteins that are enriched for GO terms associated to DNA packaging, histone modification and regulation of transcription. These proteins include histones like HHT1 and HTA2, and the histone chaperone NAP1 (see Figure 7). For menadione, the combined metric improved the ranks of the drivers GLN3, YAP1, and UME6. Their protein interactors were statistically enriched in processes like “chromatin assembly and disassembly”, “histone modification” and “nuclear import.” Some examples of the interactors falling into these categories are shown in Supplementary Figure 8. For instance, the protein RSC3 is a member of a chromatin-remodeling complex and regulates stress response and ribosomal genes. CRM1 is involved in exporting mRNA and protein molecules from the nucleus. Supplementary Figure 8 also shows the driver ranks for diamide and DTT. For diamide, the combined metric improved the ranks of seven drivers: PDR1, FKH2, GCR2, STB5, YAP1, NRG1, and UME6. The interactors of these drivers were enriched for chromatin organization. For DTT, the combined metric improved the ranks of two drivers: GCR2 and SKN7. There were not enough interactors in this case to find significantly enriched GO terms.

In the viral oncogene data, we focused on the following driver TFs whose ranking improved through the combined network score: WT1, TCF12, MAX, SMAD4, EBF1, NFE2L2,

RARA, and TAL2. Note that, due to the large number of cancer drivers, we analyzed only the ones that were present among the top 10% of TFs as ranked by at least one of the three metrics listed above. Figure 8 depicts all the interactions with this set of proteins. Most of the enriched GO terms with high odds ratios pertained to regulation of transcription and organism development. For example, the GO term “muscle organ development” is enriched due to the presence of four proteins including ID3, a transcriptional inhibitor that can modulate cell differentiation, and UNC45A, which is part of the progesterone signaling pathway and is needed for cell proliferation. Enrichment of the GO term “cytoplasmic sequestering of transcription factor” arises from SRI, a gene that binds calcium and can regulate the activity of calcium channels, and MXI1, a protein that competes with MAX to bind MYC and inhibit its function.

Taken together, these results suggest that driver TFs tend to be ranked higher when integrating the PPI network degree because they are more often regulated by proteins like nuclear exporters, transcriptional repressors, or chromatin remodelers, and in humans, they may interact with signaling molecules that carry information about the environment (e.g. calcium or hormone levels).

Supplementary Figure Captions

Supplementary Figure 1 Combined network score improves enrichment in driver genes in a manner robust to data sampling. ROC curves show the performance of the three network measures for the 53 yeast strains most sensitive to rapamycin. P-values are computed using Kolmogorov-Smirnov test. AUC = area under the curve. Bar graphs show odds ratio for the overlap between driver TFs and the top 20% TFs ranked by each measure.

Supplementary Figure 2 Driver TFs have high degree in PANDA regulatory network associated with rapamycin response. (A) Receiver-operator characteristic (ROC) curves showing performance of two different measures – degree in PANDA transcriptional network and differential expression after addition of rapamycin for 50 minutes – in identifying driver TFs. P-values are computed using Wilcoxon test. (B) Bar graphs show the odds ratio for the overlap between driver TFs and the top 10, 20 and 30% of TFs ranked by the same two measures. (C) Transcriptional network inferred by PANDA in rapamycin-perturbed yeast. Only TFs and their interactions are shown. Red nodes denote rapamycin driver genes. The size of the node is proportional to its degree in the full network, including all target genes.

Supplementary Figure 3 Combining degree in transcriptional network and yeast two-hybrid (Y2H) protein interaction network improves enrichment in driver genes. Figure depicts ROC curves showing performance of three different measures – degree in transcriptional network, degree in Y2H protein interaction network, and combined network score – in identifying driver TFs. P-values are computed using Kolmogorov-Smirnov test. AUC = area under the curve. Bar graphs show odds ratio for the overlap between driver TFs and the top 20% of TFs ranked by each of the three measures.

Supplementary Figure 4 For rapamycin response in yeast, combining differential expression and protein interaction network results in a similar increase in enrichment for drivers as combining transcriptional network with the protein interaction network. The ROC curves show the performance of five network measures. Bar graphs show odds ratio for the overlap between driver TFs and the top 20% TFs ranked by each measure.

Supplementary Figure 5 Among stress conditions where GMIT network is not enriched for drivers, protein interaction network can improve enrichment. Figure depicts ROC curves

comparing performance of degree in GMIT transcriptional network, degree in protein interaction network, and combined score, for all other growth conditions. P-values are computed using Kolmogorov-Smirnov test and AUC denotes area under the curve.

Supplementary Figure 6 Among stress conditions where PANDA network is not enriched for drivers, protein interaction network can improve enrichment. Figure depicts ROC curves comparing performance of degree in PANDA transcriptional network, degree in protein interaction network, and combined score, for all other growth conditions. P-values are computed using Kolmogorov-Smirnov test and AUC denotes area under the curve.

Supplementary Figure 7 For viral oncogene-perturbed human fibroblasts, combining differential expression with protein interaction data tends to decrease enrichment in drivers, as compared with combining the transcriptional and protein interaction networks. Large ROC curves show the performance of five network measures. Bar graphs show odds ratio for the overlap between driver TFs and the top 10% TFs ranked by each measure. Small ROC curves below show just the two combined scores for better visual comparison.

Supplementary Figure 8 The driver TFs with high combined network score interact with proteins enriched for chromatin assembly, histone modification and nuclear transport. Heatmaps show the ranks of all driver TFs according to either the transcriptional network degree, the protein interaction degree, or the combined network score, in the cases of menadione, DTT and diamide response in yeast. Network depicts all direct protein interactors of the menadione driver TFs that had a higher rank in the combined network score than in either individual network alone. Edges represent evidence of direct protein interaction from yeast two-hybrid experiments.

Supp Figure 1

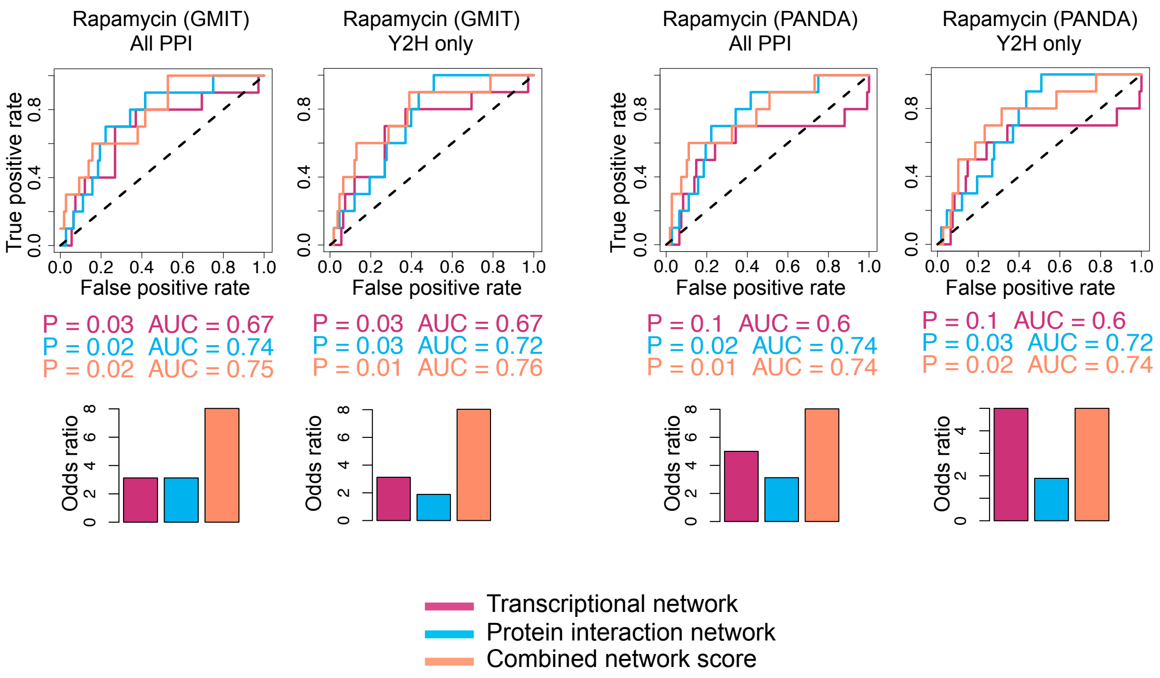

Supp Figure 2

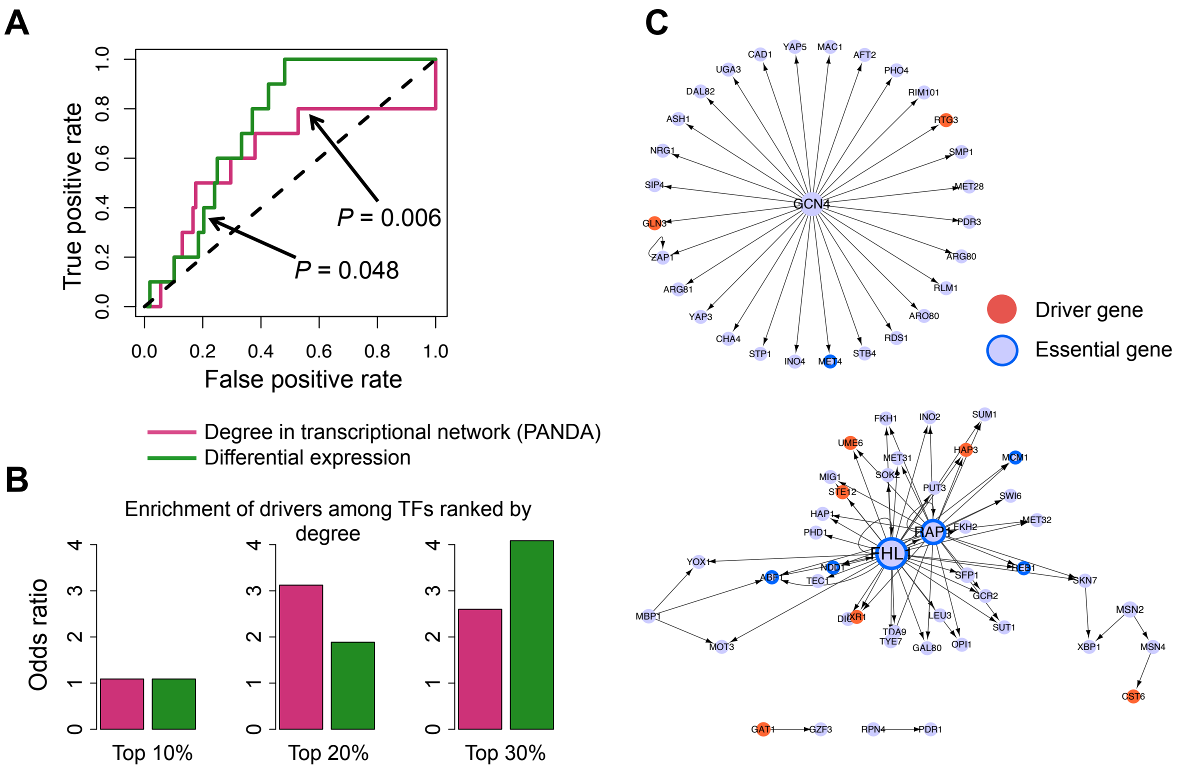

Supp Figure 3

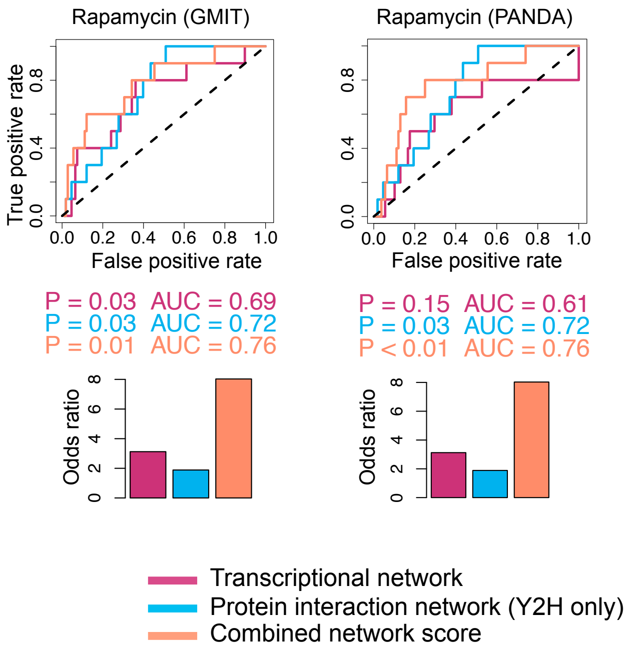

Supp Figure 4

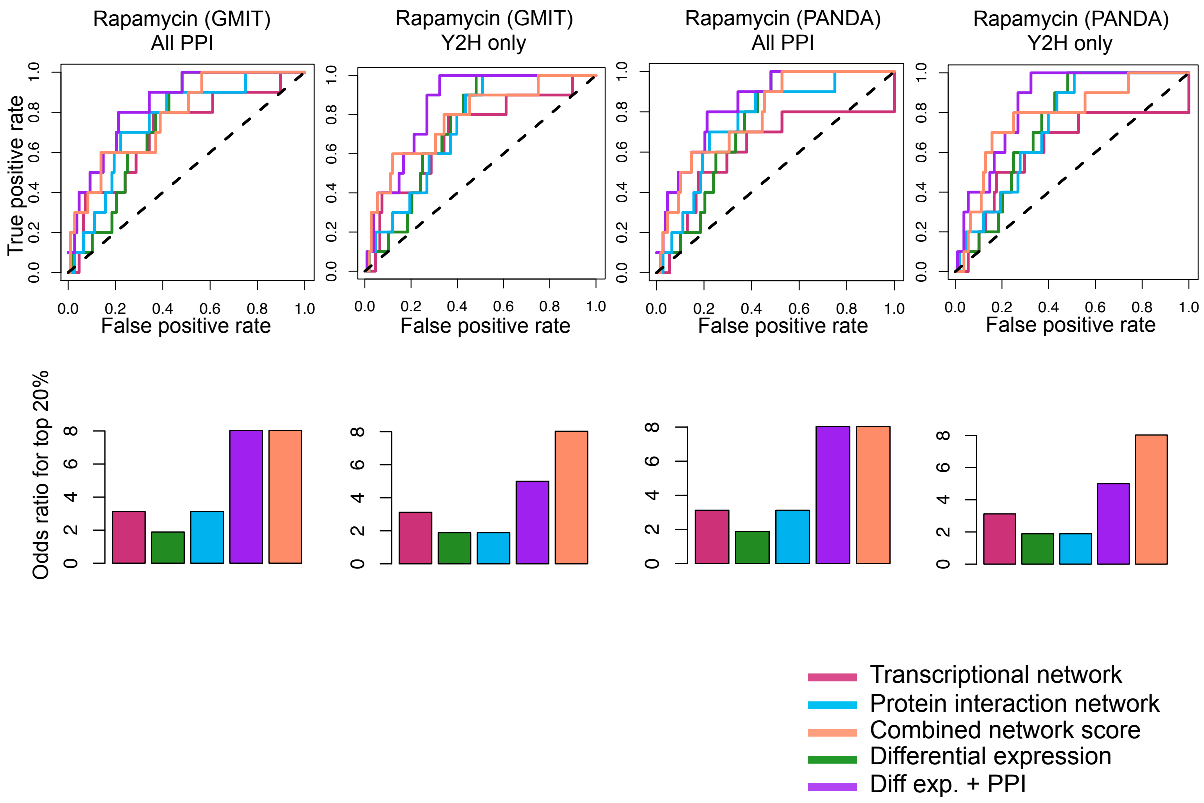

Supp Figure 5

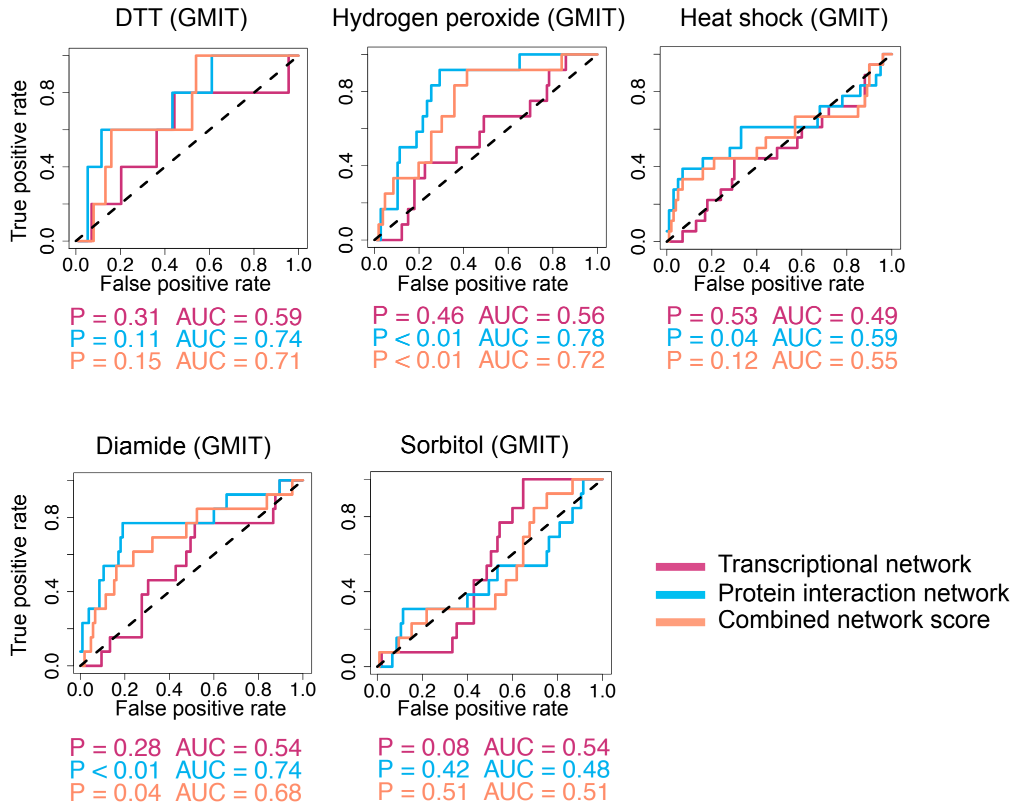

Supp Figure 6

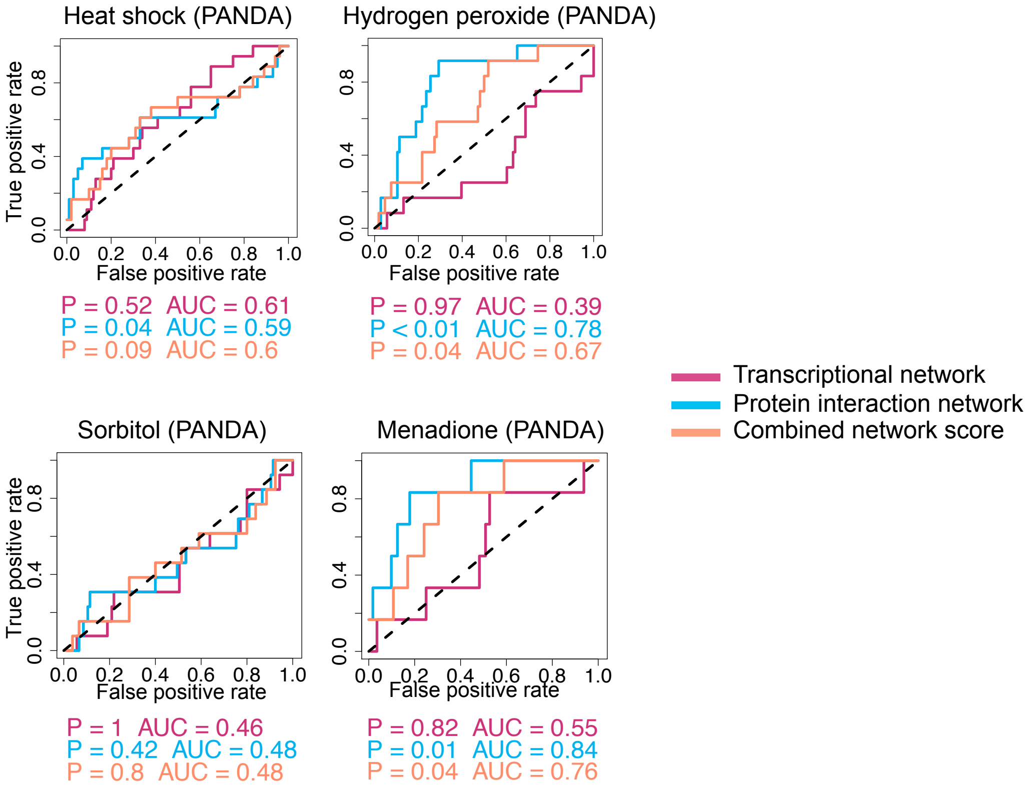

Supp Figure 7

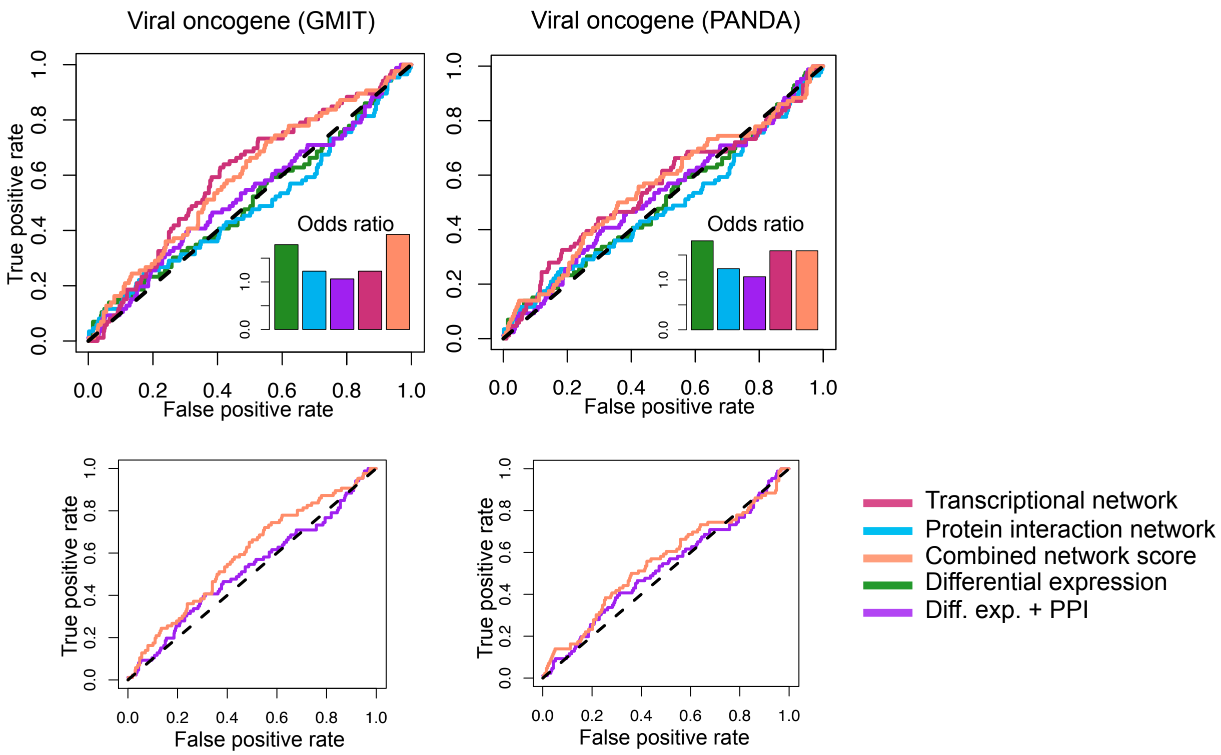

Supp Figure 8

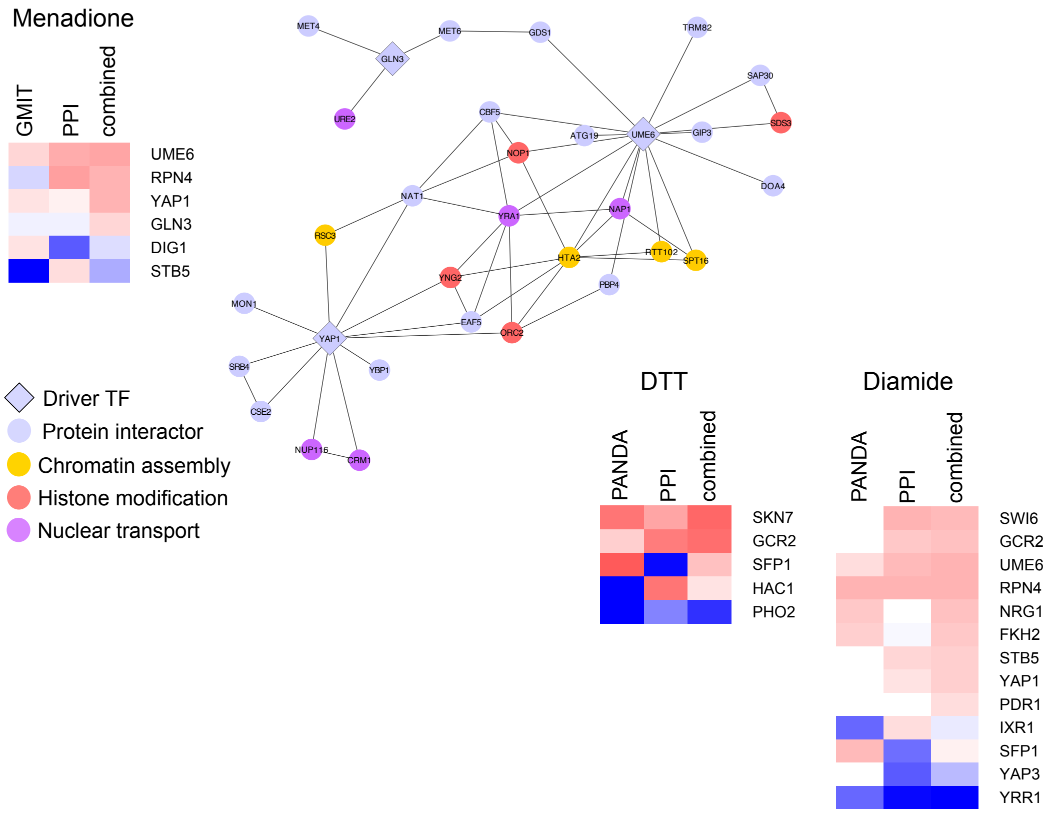

Supplement: Additional file 1: Supplementary Text and Figures. — Supplementary text describes (a) the robustness of the results to the growth rate cutoff for yeast strains, (b) an analysis combining differential expression with protein interaction degree, and (c) the characterization of the protein interactors of driver TFs. Figures present results for (i) network integration for rapamycin data with alternative growth cutoff, (ii) PANDA analysis of rapamycin expression data, (iii) rapamycin network integration using only yeast two-hybrid data, (iv) results of combining differential expression with PPI degree, (v) ROC curves for network integration in multiple yeast conditions, and (vi) the rank characteristics and local PPI neighborhoods of driver TFs for menadione, DTT and diamide. (PDF 1304 kb) [file 12918_2015_228_MOESM1_ESM.pdf]
